# Pituitary Adenylate Cyclase-Activating Polypeptide: A Promising Neuroprotective Peptide in Stroke

**DOI:** 10.14336/AD.2020.0626

**Published:** 2020-12-01

**Authors:** Yuanjian Fang, Reng Ren, Hui Shi, Lei Huang, Cameron Lenahan, Qin Lu, Lihui Tang, Yi Huang, Jiping Tang, Jianmin Zhang, John H Zhang

**Affiliations:** ^1^Department of Neurosurgery, The Second Affiliated Hospital, School of Medicine, Zhejiang University, Hangzhou, Zhejiang, China.; ^2^Department of Neurosurgery, Yongchuan Hospital, Chongqing Medical University, Chongqing, China.; ^3^Department of Neurosurgery, Loma Linda University, Loma Linda, CA, USA.; ^4^Department of Physiology and Pharmacology, Loma Linda University, Loma Linda, CA, USA.; ^5^Burrell College of Osteopathic Medicine, Las Cruces, NM, USA.; ^6^Department of Neurosurgery, Sir Run Run Shaw Hospital, Zhejiang University, School of Medicine, Hangzhou, Zhejiang, China.; ^7^Department of Anesthesiology, Loma Linda University, Loma Linda, CA, USA.

**Keywords:** stroke, pituitary adenylate cyclase activating polypeptide, cerebral ischemia, intracerebral hemorrhage, subarachnoid hemorrhage

## Abstract

The search for viable, effective treatments for acute stroke continues to be a global priority due to the high mortality and morbidity. Current therapeutic treatments have limited effects, making the search for new treatments imperative. Pituitary adenylate cyclase-activating polypeptide (PACAP) is a well-established cytoprotective neuropeptide that participates in diverse neural physiological and pathological activities, such as neuronal proliferation, differentiation, and migration, as well as neuroprotection. It is considered a promising treatment in numerous neurological diseases. Thus, PACAP bears potential as a new therapeutic strategy for stroke treatment. Herein, we provide an overview pertaining to the current knowledge of PACAP, its receptors, and its potential neuroprotective role in the setting of stroke, as well as various mechanisms of neuroprotection involving ionic homeostasis, excitotoxicity, cell edema, oxidative stress, inflammation, and cell death, as well as the route of PACAP administration.

Stroke is a catastrophic disease associated with high mortality and morbidity in humans [1]. Stroke is traditionally divided into two subtypes: hemorrhagic stroke and ischemic stroke [2]. Despite the tremendous efforts emphasizing the prevention, treatment, and recovery of stroke, the mortality remains high [1]. Numerous pathophysiological mechanisms participate in brain injury following stroke, including excitotoxicity, ionic homeostatic disorder, oxidative stress, cell edema, cell death, and blood-brain barrier (BBB) dysfunction [3, 4]. Given the complexity of these processes, it is difficult to find potent drugs to treat stroke patients. Furthermore, the time required to progress from pre-clinical studies to clinical trials, and lastly, to clinical treatment, is extensive [5]. Despite these obstacles, stroke research is still encouraged, which may shed light on novel therapeutic treatments for stroke patients.

Pituitary adenylate cyclase-activating polypeptide (PACAP) is a neuropeptide with a structure that has remained evolutionarily conserved since the protochordate. It was initially discovered in hypothalamic tissue three decades ago [6, 7], and is considered as a member of the vasoactive intestinal peptide (VIP)/glucagon/secretin family due to its high homology with VIP on the N-terminus amino acid sequence [7]. Until now, PACAP has been revealed to have widespread occurrence and diverse biological effects in mammalian peripheral and central nervous systems (CNS) [8, 9]. Accumulating evidence indicates that PACAP participates in neuronal proliferation, differentiation, and migration [10]. Recently, it was found to function as a potential effective therapeutic for various chronic nervous system disorders, such as post-traumatic stress disorder [11], multiple sclerosis [12], migraine [13], and even dry eye syndrome[14]. Furthermore, it was generally upregulated in several types of acute pathological conditions, including cerebral ischemia [15], intracerebral hemorrhage (ICH) [16], subarachnoid hemorrhage (SAH) [17], and traumatic brain injury [18]. Knockdown of PACAP or administration of exogenous PACAP in the animal model of ischemia stroke demonstrated the neuroprotection provided by PACAP, resulting in the attenuation of neurological deficits and pathological changes [19]. The clinical studies also indicated that the higher plasma PACAP level was significantly related to the improved prognosis of SAH and ICH patients [16, 17].

The aim of this review is to provide insight into the role of PACAP in stroke. Briefly, we first review the current knowledge of PACAP and its receptors within the nervous system. Then, we discuss the current known and potential neuroprotective mechanisms of PACAP against stroke in regard to ionic homeostatic, excitotoxicity, cell edema, oxidative stress, inflammation, cell death, and blood-brain barrier (BBB) dysfunction. Lastly, we summarize the various approaches of PACAP administration in the animal model of stroke.

## PACAP and receptors

PACAP is a neuropeptide found in several brain regions of rodents (e.g. hypothalamus, anterior pituitary, hippocampus, superior colliculus [20], suprachiasmatic, paraventricular and periventricular hypothalamic nuclei [21], trigeminal nucleus caudalis, and cervical spinal cord [22]). Encoded by the ADCYAP1 gene located on chromosome 18, PACAP has two multiple transcript variants, PACAP27 and PACAP38, which are named according to the number of amino acids at the N-terminus [23]. PACAP38 is considered the predominantly expressed isoform in mammalian tissues compared with PACAP27 [8].

As a member of the VIP/secretin/growth hormone-releasing hormone family, PACAP has a 68% sequence homology with VIP, and 37% with secretin [24]. Due to the increased homology between PACAP and VIP, three different G protein-coupled receptors are shared, which mediate the actions of VIP and PACAP, including PAC1 (encoded by Adcyap1r1, also named PAC1-R), VPAC1, and VPAC2 (encoded by Vipr1 and Vipr2, also known as VPAC1-R and VPAC2-R). Because PAC1 has a 100-fold selectivity for PACAP27 and PACAP38 over VIP, it functions as the primary receptor of PACAP [9]. In the rat brain, PAC1 is widely expressed in the cortex, hippocampus, olfactory bulb, brainstem, hypothalamus, and cerebellum [25, 26]. While VPAC1 is mainly present in the cerebral cortex and hippocampus, VPAC2 is found primarily in the thalamus, hypothalamus, hippocampus, central nucleus of amygdala, and brainstem [27, 28].

**Figure 1.**
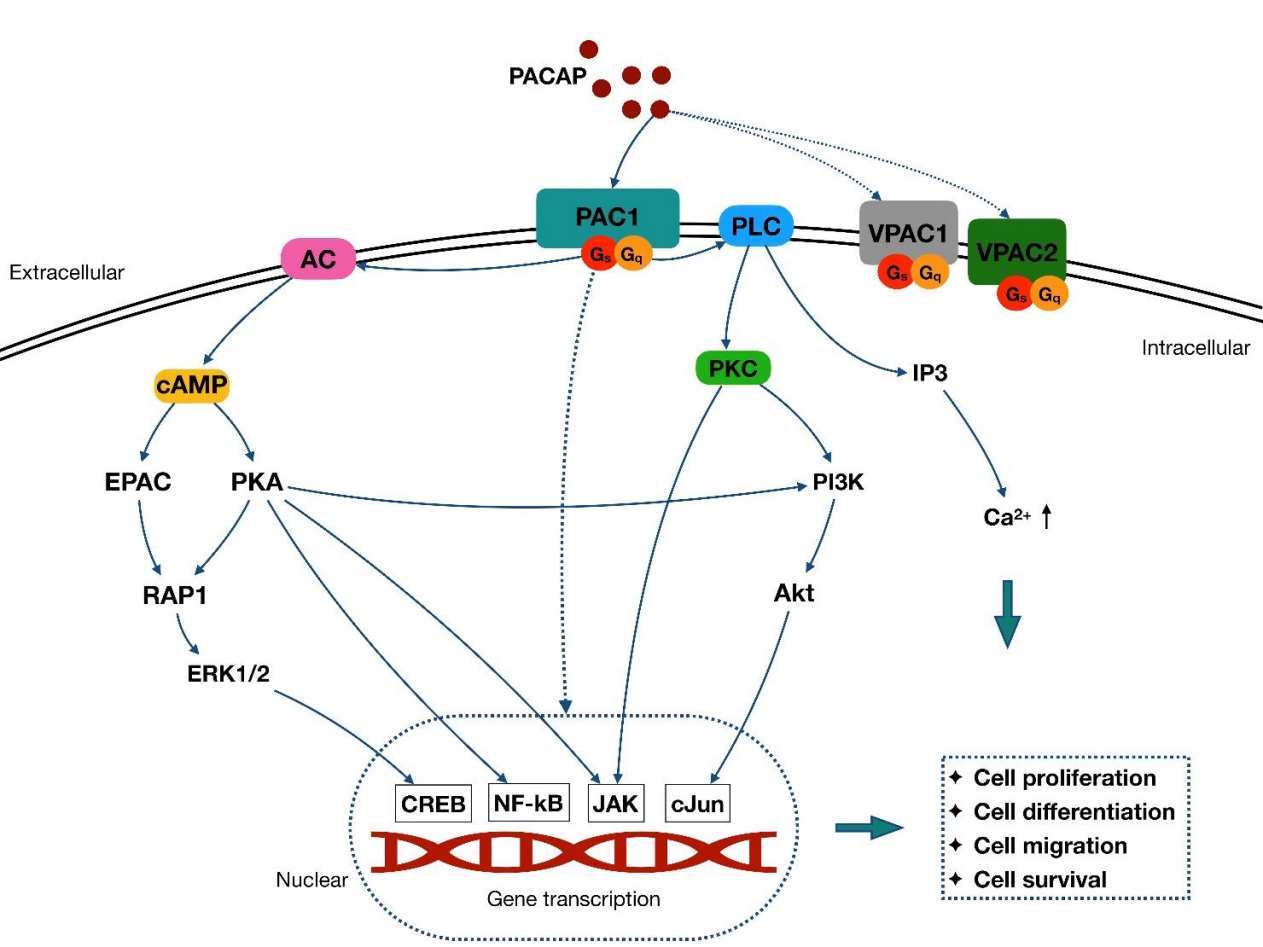
Schematic pathway of PACAP signaling cascades. PACAP exerts function via activation of three different G-protein coupled receptors: PAC1, VPAC1, and VPAC2. The PAC1 has a 100-fold selectivity for PACAP over VIP, contributing to its role as the main functional receptor of PACAP. Each PACAP receptor is coupled primarily to Gs or Gq, which stimulate AC and PLC activation. In the AC-involved signaling pathway, AC accelerates ATP conversion to cAMP, which then prompts PKA phosphorylation and activation of EPAC pathway. In contrast, activation of PLC boosts the PKC pathway, and the IP3 activation to increase the intracellular Ca^2+^. Both PLC and AC/cAMP signaling pathways are related to the PACAP function by mediating downstream targets, such as the MAPKs family and the PI3K/Akt pathway. These pathways further mediate cell proliferation, differentiation migration, and survival through several nuclear genes, such as CREB, NF-kB, JAK and cJun. Besides, these downstream signaling pathways also appear to be directly activated by PAC1 and are paralleled with PLC and AC/cAMP pathway in some cells.

Each PACAP receptor is coupled primarily to Gs or Gq, which stimulate adenylyl cyclase (AC) and phospholipase C (PLC) activation. In the AC-involved signaling pathway, AC accelerates ATP conversion to cyclic adenosine monophosphate (cAMP), and then prompts protein kinase A (PKA) phosphorylation (primary) [29] and other cAMP downstream (secondary), such as exchange proteins activated by cAMP (EPAC) [30]. In contrast, PLC activation boosts both the protein kinase C (PKC) function [31, 32] and 1,4,5 inositol triphosphate (IP3) [33]. Furthermore, the PLC and AC/cAMP signaling pathway could mediate downstream targets, such as ras-associated protein-1 (RAP1)[34], MAPKs [35-38], NF-kB [39, 40], janus kinase (JAK) [41, 42], cJun [43, 44], and phosphatidylinositol 3-kinase (PI3K) [45]. In parallel with the PLC and AC/cAMP pathway, these downstream signaling pathways may also be directly activated by PAC1 in some cells [29, 46, 47] (Fig. 1).

## Function of PACAP in nervous system

PACAP functions as a master regulator of the stress response, maintaining homeostasis within the central and peripheral nervous systems. In general, the functional roles of PACAP can be categorized as neuronal development, neuromodulation, and neuroprotection [10, 48].

PACAP participates in the development of neuronal and glial cells [49, 50]. It mediates neuronal proliferation, migration, differentiation [51], and axonal growth and elongation [52]. As reviewed by Rivnyak *et al*. [10], these PACAP-involved processes are highly related to 1) several cell survival genes, such as the MAPK family (with downstream CREB) and calcium calmodulin pathway genes [8, 10]; 2) cell cycle regulator family, cyclin and mitogenic related factors, such as janus kinase 1 (JAK1) and the signal transducer and activator of transcription 3 (STAT3) [53, 54]; and 3) cell differentiation- and migration-related factors, including transforming growth factor beta (TGF-β), fibroblast growth factor (FGF21), nerve growth factor-induced protein A (EGR1), brain-derived neurotrophic factor (BDNF), insulin-like growth factor (IGF1), and bone morphogenic protein receptor type 2 [10]. Additionally, PACAP stimulates the myelination process by facilitating the proliferation of oligodendrocytes, while delaying their maturation [55]. Moreover, it promotes astrogliogenesis and differentiation only in non-carcinoma circumstances [56, 57].

PACAP is an important neurotransmitter and neuromodulator, functioning in behavioral processes, cognitive performance, emotional formation, and homeostatic regulation [10, 29, 48]. PACAP and the receptor, PAC1, are highly expressed in the hypothalamic nuclei, which is important for appetite and satiety [58, 59]. Studies showed that PACAP administration negatively affected food intake in rodents [60, 61]. This effect may be potentially integrated with the activity of the satiety-promoting neuropeptides, proopiomelanocortin and α-melanocyte-stimulating hormone (αMSH), which are co-expressed with PACAP in the ventromedial, arcuate, and paraventricular nuclei of the hypothalamus [62, 63]. Similarly, PACAP is also co-expressed with leptin in the ventromedial nucleus, which then regulates thermogenesis and feeding [59]. The thermogenesis of PACAP is also regulated by brown adipose tissue, uncoupling protein 1, and neurons within the sympathetic nervous system [64-66]. With the regulation of the sympathetic nervous system, PACAP is also involved in glycemic and lipocatabolic metabolism, which contributes to energy maintenance [66]. In terms of cognitive performance, PACAP participated in the hippocampal glutamatergic synaptic transmission, plasticity, and learning [67]. Accumulating evidence suggested that the reduced PACAP level was related to the development of cognitive impair in Alzheimer’s disease, Parkinson’s disease, and Huntington’s disease [67].

PACAP is characterized as a sensory peptide, which further modulates emotional formation. It is expressed on the sensory dorsal root and trigeminal ganglion neurons, it participates in pain conduction and emotional formation [68, 69]. Moreover, the role of PACAP in the peripheral pain circuits was widely acknowledged [70]. PACAP induces chronic pain by participating in pain signaling conduction, which is terminated in the lateral capsular division of the amygdala and limbic nucleus, further eliciting emotional responses, such as stress, anxiety, and fear [71, 72]. Based on these characteristics, PACAP is considered an important part of the pathogenic mechanism involved in post-traumatic stress disorder and migraines [11, 73].

Regarding neuroprotection, PACAP is engaged in endogenous protective mechanisms in the acute and chronic cell stress-response [10]. PACAP is generally upregulated in the CNS, and acts against neural damage from several harmful agents, such as kainic acid [74], ethanol, nicotine [75], oxidative stress-related agents [76], glucotoxicity, hypoglycemia-induced toxicity [77], beta-amyloid peptide [78], and thrombin [79]. Administration of exogenous PACAP improved the outcomes in a variety of animal models, including traumatic brain injury[80], stroke [81], retinal ischemia [82], spinal cord injury [83], Parkinson’s disease [84], Huntington’s disease [85], and spinobulbar muscular atrophy [86].

## PACAP and PAC1 receptor changes after stroke

PAC1 has been widely considered the main receptor of PACAP. Therefore, PACAP and PAC1 expression were often studied together in the setting of rodent stroke models. The brain levels of PACAP and PAC1 expression varied in the different regions and types of ischemic stroke [19]. Following global ischemia, PACAP was decreased in the first 24h, but increased 2-7 days after transient global ischemia in the murine hippocampus [87-89]. The upregulated PACAP may be secreted from neuronal stem cells in the subgranular zone, and from mature neurons in the hilus of the mice hippocampus [88]. Similarly, the PAC1 expression decreased in the hippocampus granule cells within the first 3 days after global ischemia [89], but increased 3-28 days after global ischemia in hippocampal astrocytes [90, 91]. These results indicated that the PACAP/PAC1 signaling pathway may play an important role in the hippocampal neurorepair process in the subacute and chronic stages after global ischemia. The protein kinase RNA-like endoplasmic reticulum kinase receptor response occurs within minutes to hours of unfolded protein response activation, which may account for the reduced PAC1 expression in the acute phase. This was shown to suppress PAC1 expression in the mouse neuroblastoma cell line [92].

Conversely, PACAP and PAC1 increased immediately in the cortex after focal cerebral ischemia induced by middle cerebral artery occlusion (MCAO) [93, 94]. PACAP and PAC1 were mainly expressed on cortical astrocytes, neurons, and endothelial cells [90, 93, 95]. The HIF-1α activation after ischemia may increase the expression of PACAP signaling in the cortex. It promoted PACAP/PAC1 expression and homing of bone marrow-derived cells to the hypoxic area. HIF-1α is considered a double-sided transcription factor in different hypoxic conditions, which may explain the differences of PACAP and PAC1 expression that occur between global and focal ischemia [93].

**Figure 2.**
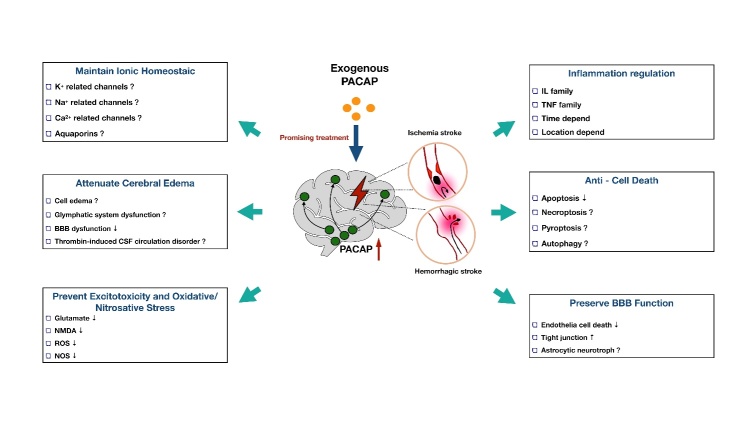
Current and proposed function of PACAP in different pathophysiology after stroke. PACAP is a promising neuroprotective peptide with great potential to mediate ionic homeostasis, cerebral edema formation, cytotoxicity, oxidative/nitrosative stress, inflammation, cell death, and BBB function after stroke.

Although ischemic conditions change region-specific crossing, exogenous PACAP treatment still exerted sufficient neuroprotection to the ischemic brain. A plethora of evidence has proven that exogenous PACAP reduces brain injury in both focal and global ischemia models [96-99]. However, the current research regarding the role of PACAP on hemorrhagic stroke is limited. Only two papers have indicated that the higher plasma PACAP level was related to a better outcome in SAH and ICH patients [16, 17]. Considering that there is plenty of overlap pertaining to the pathophysiological mechanisms of hemorrhagic and ischemic stroke, we boldly proposed that the PACAP treatments will attenuate both types of stroke by exerting the following neuroprotective effects: anti-apoptosis [43], anti-inflammation [46], anti-oxidative stress [100], anti-excitotoxicity [101], ionic equilibrium maintenance [102], and vascular protection [103] (Fig. 2). In the following sections, we will discuss how PACAP potentially regulates these molecular pathological processes in stroke.

## PACAP and potential mechanism of neuroprotection in stroke

### PACAP and ionic homeostatic disorder

Ion redistribution begins immediately after stroke and is associated with several pathological changes, including cell edema [104] and excitotoxicity [105]. Numerous ionic transporters, such as Na^+^-K^+^ATPase and Ca^2+^- ATPase, and ion channels and cotransporters, such as the Na^+^-K^+^-Cl^-^ co-transporter (NKCC1) and the Na^+^/Ca^2+^ exchanger, increases activity after stroke [104]. The influx of ions generates a transmembrane osmotic gradient that favors the influx of water and leads to cellular edema [106]. Dysfunction of calcium homeostatic machineries may exacerbate calcium overload and contribute to excitotoxicity companied with glutamate [107].

PACAP is a promising ionic homeostatic regulator. As reviewed, PACAP was involved in the modulation of several intrinsic ion or water channels responsible for mediating a variety of physiological activities, such as voltage-gated potassium channels [108], T-type calcium channels [109], voltage-gated sodium channels [110], and calcium-dependent potassium channels [30], which mediate neuronal excitability; hyperpolarization-activated cyclic nucleotide gated channels, which mediate spike frequency and pacemaking in rhythmic firing neurons [111], and aquaporins (AQP), which mediate edema [112]. However, the effects of PACAP on these channels were completely regulated by different pathways. Phosphorylation via PKA, PKC, or ERK is considered a common mechanism of regulating channel activity [102]. In addition, PACAP could also increase intracellular calcium concentrations via activation of EPAC, which may promote calcium-dependent astrocytic differentiation of neural precursor cells [113].

In a study of a rat global cerebral ischemia model, it was found that the sodium contents increased, but the potassium contents decreased in the ischemic brain. Treatment with an intracerebroventricular injection of PACAP38 attenuated this ionic change in brain tissue [114]. Therefore, ion/water channels could be a potential therapeutic target of PACAP, which may mediate ion homeostatic disorder-induced cellular edema and excitotoxity in stroke.

### PACAP and cerebral edema

Cerebral edema happens inevitably following stroke. It is initiated immediately after losing the cellular ionic redistribution, and largely depends upon the activity of ion/water channels or transporters on the neurovascular unit [115]. Changes in ion and water distribution generate abnormal osmotic forces that manifest as cerebral edema, further inducing or aggravating hypoxia, inflammation, cell death, and BBB disruption [104].

Exogenous PACAP38 treatment after ischemic stroke was proven to attenuate the increase in brain water content following transient global ischemia in rats via activation of PAC1 [114]. The brain edema volumes were also decreased in a mouse model of permanent MCAO (pMCAO) after exogenous PACAP38 treatment [116]. As for SAH, we have found that PACAP38 treatment decreased the brain water content and intracerebral pressure increasement after SAH in rats (unpublished data).

It should be mentioned that PACAP is a controversial vasodilator, which mediates the dermal edema formation in mice and rabbits [117, 118]. In a study focusing on the vasodilatory effect of PACAP, it was found that dosages higher than 50nmol/kg (intravenous injection) would cause a long-lasting potent decline of arterial blood pressure, regional cerebral blood flow, and cerebral oxygen content in mice. Conversely, the low dosage would be safe for neuroprotective treatments of the brain [119]. However, there is no difference in changes of cerebral blood flow between the PACAP knockout and the PACAP wide-type mice during ischemia [116].

BBB disruption, glymphatic system dysfunction, and cerebrospinal fluid (CSF) circulation are mechanisms involved in edema formation [104, 120]. After hemorrhagic stroke, thrombin and platelets are detrimental substances involved in the pathological change of the second brain injury that leads to cell death, inflammation, and CSF circulation blockage [121, 122]. Several studies have suggested that PACAP can inhibit the platelet aggregation induced by arachidonic acid and thrombin, potentially through the cAMP-dependent pathway [122]. In view of this, we postulate that the PACAP may attenuate brain edema by inhibiting thrombin- and platelet aggregation-induced impairment of CSF circulation after hemorrhagic stroke, which is worthy of further investigation in the future.

### PACAP and excitotoxic injury

Excitotoxic injury is one of the major pathological changes after stroke, and is caused by an excess of excitatory amino acids, such as glutamate and N-Methyl-D-aspartate (NMDA) [105]. The over-accumulation of glutamate may result in a surge of calcium influx, causing high intracellular calcium concentrations, further leading to excitatory neuronal damage [123]. It was first found in several neuronal cell lines, such as the rat cortical neuronal cell line [124], PC12 cell line [125], and the rat motor neuron cell line [126]. Endogenous PACAP improved cell viability after excitotoxic glutamate exposure. The endogenous PACAP mRNA levels were upregulated after glutamate exposure in the rat neuron cell line [127]. The PAC1 receptor was suggested to be involved in the anti-excitotoxic process of PACAP. Treatment with PACAP6-38, the PAC1 selective antagonist, exacerbated cell death after glutamate exposure [127]. Meanwhile, PACAP was also shown to decrease glutamate-stimulated nitric oxide (NO) production in PC12 neurons, and further increased the cell viability. However, treatment with a PKA or PKC inhibitor abolished the downregulation of NO production. Moreover, the ERK inhibitor had no effect on this pathway [128].

NMDA also leads to excitotoxic injury after stroke. In the oxygen-glucose deprivation/reperfusion model of primary rat neural cells (40% neurons and 60% astrocytes), PACAP attenuated cell death by modulating the expression levels of NMDA receptor subunits. PACAP increased the protein expression levels of the GluN1 subunit GluN2C (pro-survival), and decreased that of the GluN2B and GluN2D subunits (pro-cell death) [129]. Moreover, direct local treatment by non-vasoactive doses of PACAP (both PACAP27 and 38) prevented the post-ischemic cerebral vasodilation caused by NMDA in neonatal pigs [130].

Glutamate transporter-1 (GLT-1) is responsible for the majority of glutamate transportation to astrocytes, and plays an important role in preventing overstimulation of glutamate receptors [131]. The glutamate is further degraded by glutamine synthetase (GS) in astrocytes [132]. Exposure of cultured astroglia to PACAP-38 was found to be capable of upregulating the expression of GLT-1 and GS, which promoted the glutamate uptake via a PAC1-dependent manner. The PKA and PKC signaling was also involved in this pathway [132]. Additionally, PACAP-38 was proven to participate in the neuroprotection of Buyang Huanwu decoction (a Chinese herbal medicine). It conveys neuroprotection by upregulating the expression of GLT-1 and GS in the rat MCAO focal cerebral ischemia model [133]. Collectively, PACAP may exert neuroprotection through its potent anti-excitotoxicity effects after stroke. Additionally, *in vivo* studies are needed to validate the *in vitro* findings of PACAP, and to elucidate the potential downstream signaling pathways involved.

### PACAP and oxidative and nitrosative stress

The oxidative and nitrosative stresses are characterized as an imbalance between the systemic manifestation of reactive oxygen/nitrogen species (ROS/RNS) and a biological system's ability to detoxify the ROS/RNS, which causes cell damage. In the sense, the oxidative and nitrosative stresses are downstream consequences of excitotoxicity. Excitotoxicity could result in a rise in secondary messenger systems, which would further trigger the enzymatic generation of free radicals, including cyclooxygenase and NOS after stroke [123, 134].

PACAP has been shown to attenuate oxidative stress-induced damage in several types of neural cells. In a neuronal culture, PACAP increases cell viability in cerebellar granule neurons that were exposed to H_2_O_2_ through a PAC1-, cAMP-, or MAPK-dependent pathway [76], and activated aconitase, a survival mitochondrial enzyme in response to oxidative stress [135]. PACAP also promotes the expression of several detoxifying kinases at the gene or protein level, such as peroxiredoxin 5, thioredoxin reductase [136], glutathione, and superoxide dismutase [137]. In glial cell culture, PACAP-38 reduces the production of microglia-derived ROS after MPP^+^ stimulation [138], suggesting that PACAP is also involved in microglia-mediated oxidative stress. PACAP-38 activated PAC1, as well as the PKA, PKC, and MAPKs signaling pathways, which further inhibit the accumulation of ROS, and increases anti-oxidant enzymes, such as superoxide dismutase and catalase antioxidant enzyme in a cultured rat astrocytic cell line [138]. Moreover, PACAP also showed a pro-survival effect against glucose deprivation- and oxidative stress-induced junctional damage in the microvascular brain endothelial cell line [138]. The protective effects of PACAP have also been shown against lipopolysaccharide (LPS)-induced release of nitric oxide (NO) and lactate dehydrogenase [139], as well as NO-releasing neurotoxin sodium nitroprusside in primary cortical neuronal cultures [79].

Based on the current evidence regarding the pro-survival effects of PACAP in neural cells exposed to oxidative and nitrosative stress, the PACAP may be a promising anti-oxidative and anti-nitrosative treatment in animal models of stroke. However, limited studies have evaluated the effect of PACAP on oxidative and nitrosative stress after stroke *in vivo*. In a rat global ischemia model, PACAP-38 was proven to increase the hippocampal protein and mRNA level of 5′-acting AP endonuclease 1 (APE1) activity and decrease DNA damage and neuronal death in the first day after reperfusion. The APE1, also known as redox-effector factor 1, is a critical protein for base-excision repair after oxidative DNA damage[140]. The potential mechanism is that PACAP-38 upregulated PKA- and p38-dependent phosphorylation of CREB and activating transcription factor (ATF) 2, leading to transactivation of the APE1 promoter [141]. Nevertheless, future studies are necessary to validate the anti-oxidative mechanism of PACAP in animal models of stroke.

### PACAP and neuroinflammation

The immune-mediated inflammatory response started immediately after the stimulation of several damage-associated molecular pattern molecules (DAMPs) produced by ischemia or hemorrhage [142, 143]. Inflammation plays a double-edged role in the pathogenesis of stroke and other forms of brain injury, as it not only exacerbates secondary brain injury in the acute stage of stroke, but also beneficially contributes to brain recovery after stroke [144]. It has been proven that PACAP is involved in a broad range of inflammatory responses, resulting in either anti-inflammatory or pro-inflammatory effects [145].

The current knowledge of PACAP on neural inflammation after stroke is mainly focused on the microglial and astrocytic response. The receptors of PAC1 and VPAC1 are expressed on both cultured astrocytes and microglia [146]. Pathological microglial activation is considered a pro-inflammatory source of neuronal damage after stroke, which initiates tumor necrosis factor (TNF)-α, interleukin (IL)-1β, IL-6, IL-12, and nitric oxide (NO) cascade in the brain [147]. Delgado *et al*. [148] first found that PACAP38 inhibited the inflammatory mediators in microglial cell cultures through a cAMP-dependent intracellular pathway, which also reduced p65 nuclear translocation and NF-κB DNA binding [148]. They also showed that this anti-inflammatory effect of PACAP on activated microglia worked through inhibition of the MAPK cascades (MEKK1/MEK4/Jun N-terminal kinase (JNK)), which reduced the phosphorylation of c-Jun, and further inhibited the transcription of pro-inflammatory factors [149]. Moreover, another *in vivo* study showed that PACAP attenuates hypoxia-induced inflammatory activation of microglia by reducing NO and TNF-α, and protects co-cultured PC12 cells from microglial neurotoxicity through the MAPK p38 pathway [150]. However, it is notable that PACAP’s effect on interleukin secretion in the microglia cell culture was contrary to the astrocyte cell line. PACAP significantly increased pro-inflammatory IL-6 levels in a rat primary astrocyte cell culture [151].

Overall, the MAPK family was involved in the inflammatory mediation of PACAP after stroke. In the rat global ischemia-reperfusion model, the MAPK family, including ERK, JNK, stress-activated protein kinase (SAPK), and P38 was increased from 3h in the hippocampus after ischemia. The pre-intracere-broventricular injection of IL-6 inhibited the activation of JNK and ERK 24h after global ischemia, which proposed that the neuroprotection of IL-6 was exerted in the late phase after 24h [43]. It was also found that the PAC1 was upregulated along with IL-6 at only 3 to 7 days in hippocampal astrocytes rather than microglia after ischemia. In addition, pre-administration of low dosage PACAP38 (intracerebroventricular injection, 1pmol/mice) could increase IL-6 [152] and further protect against neuronal apoptosis [43]. The neuroprotective effect of PACAP is also absent in IL-6 KO mice [81]. The evidence suggests that PACAP promotes the late phase of astrocytic neuroinflammation in the hippocampus. However, a recent study also showed that IL-6 was expressed in microglia and peri-infarct neurons in the first 24h after focal ischemia, but the role of PACAP on IL-6 upregulation in the acute phase remains unclear [91].

The ischemic region-specific and time-dependent inflammatory response may potentially explain the divergence of PACAP effects in ischemic stroke. The effects of PACAP38 treatment on expression of cytokine genes, including IL-6, IL-22, IL1-β and IL-11, varied between the ischemic core and penumbra at 24h after pMCAO [153]. The PACAP suppressed IL-6 expression in the ischemic core and contralateral tissue, but prompted it in the ischemic penumbra, which partly suggested that the PACAP participates in neurodegeneration rather than neuroprotection in cerebral ischemia [153]. Moreover, a recent study proposed that delayed PACAP local delivery can prompt the microglial response toward a neuroprotective M2 phenotype in the late phase after ischemic stroke. The potential molecular mechanism involved the NF-κB, CREB, and the Notch/RBP-J pathway [154].

Taken together, due to the complexity of the inflammatory response after ischemic stroke, future studies should combine both *vivo* and *vitro* experiments, and at different phases to further elucidate the role of PACAP on inflammation after ischemic stroke. Besides, more studies are required that emphasize the effects on hemorrhagic stroke. Lastly, PACAP regulates inflammation by mediating cytokine/chemokine production, neutrophil mobility/migration, and T-cell differentiation in the peripheral nervous system [46, 145]. It regulates inflammation through widely expressed receptors in the immune cells. PAC1 is exclusively expressed on macrophages, while VPAC1 is expressed by all immune cells, such as macrophages, lymphocytes, and monocytes. Moreover, VPAC2 is expressed in lymphocytes and macrophages [145]. The local tissue and systemic stress response contribute to and mobilize the inflammatory response after stroke, respectively [123]. The effect of systemic administration of PACAP warrants further research in the future.

### PACAP and Cell death

Cell death, considered a terminal point of many pathophysiological changes, contributes an important role of outcome after stroke [155]. The main cell death after stroke includes apoptosis and autophagy, as well as the programmed necrosis such as necroptosis and pyroptosis [155]. Apoptosis is widely studied as a downstream target of PACAP. Numerous studies showed that PACAP has a potent anti-apoptotic effect in ischemic stroke, both *in vivo* and *vitro* [15, 129, 156, 157]. The involved mechanism may include the inhibition of the MAPK pathway, such as ERK, JNK/SAPK, and p38 signaling induced-apoptosis [44, 95]. The other classical downstream pathways of PACAP, cAMP/PKA, and PI3K/AKT pathway were also involved in the anti-apoptotic effect of PACAP in ischemic stroke [156, 157]. In addition to the direct activation of PAC1, PACAP also facilitated the neuroprotection of brain-derived neurotrophic factor (BDNF), which functions through the activation of tropomyosin-related kinase receptor type B (trkB) and neuronal growth inhibitory signaling molecules p75 (NTR) and Nogo receptor [156]. Furthermore, the tonic effect associated with astrocytes was also considered an anti-apoptotic mechanism of PACAP [15, 158].

Necroptosis and pyroptosis are two additional and important forms of programmed cell death after ischemia or hemorrhage in the brain. Both necroptosis and pyroptosis could be a result of inflammatory factors or DAMPs [4]. Recently, a paper revealed that PACAP is involved in the inhibition of necroptosis in atherosclerosis, and functions by mediating receptor interacting protein 3 (RIP3) expression, a key protein of necroptosis [159]. Additionally, PACAP can also inhibit several necroptosis and pyroptosis initiators, such as several inflammatory factors (IL family TNF family) and Toll-like receptors [39, 150]. These findings suggest that PACAP may inhibit necroptosis and pyroptosis after stroke.

The role of autophagy remains controversial in the pathological change that occurs after stroke, which may be either pro-survival or pro-death to maintain cellular homeostasis [160]. The current opinion of the effect of PACAP on autophagy after stroke also remains unclear. However, it was shown that PACAP could attenuate autophagy-induced neuronal death after hypoxic stress administration through the MAPK/ERK pathway [161]. Besides, the anti-autophagic effect was also proposed in the studies of Alzheimer’s disease [162] and atherosclerosis [159]. Interestingly, PACAP was shown to promote autophagy through the CREB pathway by preserving the acute hepatocyte death after ischemia reperfusion injury [163]. Based on the distinct roles of autophagy, we conclude that the effect of PACAP on autophagy after stroke is worthy of being further studied.

### PACAP and BBB dysfunction

The BBB is an important system responsible for maintaining the cerebral microenvironment [164]. The function and structure of BBB would be damaged after stroke, further aggravating the pathological changes. The BBB consists of the neurovascular unit, including endothelial cells, astrocytes, pericytes, neurons, and microglia [4, 165]. Among them, endothelial cells and astrocytes function as the most important components of the BBB. While PACAP receptors are expressed both on endothelial cells and astrocytes [166, 167], the effects of PACAP on BBB after stroke remains unknown. In addition, few studies have described the role of PACAP in maintaining the functionality of the BBB. Several *vitro* studies showed the protective effect of PACAP on cerebral endothelial cells. The PACAP treatment improved the barrier properties of the brain endothelium by preserving the transendothelial electrical resistance (critical marker of tight junction) after glucose deprivation and oxidative stress insults. However, it failed to prevent the apoptosis of endothelial cells [166]. In another study, the PACAP level was found to be markedly decreased in the aged endothelial cells compared with the young endothelial cell. Similarly, the PACAP treatment protected endothelial cells by promoting endothelial tube formation, and by reducing apoptosis and ROS production [168]. Further studies are needed to extend the research regarding the effect of PACAP on the neurovascular unit (not only endothelial cells but astrocytes) of BBB after stroke.

**Table 1:** The approaches of PACAP38 administration in stroke.

Year	Author	Animal	Model	Administration			
				Routes	Starting time	Duration	Best dosage
1996	Uchida D. et.al.[15]	Rats	Transient global ischemia	i.c.v. i.v.	Post-surgery Post-surgery and post-24h	Continuous	1pmol/h 16pmol/h and 160pmol/h
1998	Somogyvari-vigh A. *et.al*.[172]	Rats	Transient global ischemia	i.v.	Post-24h	Single with continuous	5nmol/kg with 160pmol/h
1998	Shioda F. et.al.[43]	Rats	Transient global ischemia	i.c.v.	Pre-48h	Continuous	1pmol/h
2000	Reglodi D. et.al.[98]	Rats	tMCAO	i.v.	Post-6h	Single with continuous	20nmol/kg with 160pmol/h
2000	Reglodi D. et.al.[99]	Rats	tMCAO	i.v.	Post-4h, 8h, 12h	Single with continuous	5nmol/rat with 160pmol/h
2002	Dohi K. et.al.[44]	Rats	tMCAO	i.c.v.	Pre-48h	Continuous	1pmol/h
2002	Yan D. et.al.[114]	Rats	Transient global ischemia	i.c.v.	Pre-15min	Single	10nmol/rat
2002	Tamas A. et.al.[96]	Rats	pMCAO	i.c.v.	Pre-surgery	Single	450pmol/rat
2002&2004	Reglodi D. et.al.[157, 171]	Rats	pMCAO	i.c.v.	Pre-surgery	Single	2μg/rat
2006	Chen Y. et.al.[97]	Mice	pMCAO	i.c.v. i.v.	Post-1h	Single	40pmol/mouse 0.75nmol/mouse
2009	Lenti L. et.al.[130]	Newborn pigs	CO2 ventilation induced transient global ischemia	Directive expose	Pre-CO2 ventilation	Continuous	10pmol/L
2010	Stetler RA. et.al.[141]	Rats	Transient global ischemia	i.c.v.	Pre-24, 12, 6h	Single	200pmol/rat
2012	Lazarovici P. et.al,[156]	Rats	tMCAO	i.v.	Post-2h	Single	30ng/kg
2012	Nakamachi T. et.al.[152]	Mice	Transient global ischemia	i.c.v.	Post-surgery	Single	1pmol/mice
2014	Hori M. et. al.[153]	Mice	pMCAO	i.c.v.	Post-surgery	Single	1pmol/mice
2014	Lin C. et al.[93]	Rats	Transient global ischemia	i.p.	Post-4h	Single	10μg/kg
2015	Brifault C. et.al.[154]	Mice	pMCAO	i.c.v.	Post-72h	Single	PACAP-producing-stem cell

Abbreviations: PACAP pituitary adenylate cyclase-activating polypeptide; tMCAO transient middle cerebral carotid occlusion; pMCAO permeant middle cerebral carotid occlusion; i.c.v intracerebroventricular injection; i.v. intravenous injection; i.p. intraperitoneal injection

## Routes of PACAP administration in stroke

Based on current animal studies, a total of four PACAP administration routes were used in stroke, including intracerebroventricular, intravenous, intraperitoneal, and direct exposure [169] (Table 1).

In spite of the evidence that PACAP can easily pass the BBB [170], intracerebroventricular administration is still considered a relatively more effective way to reach drug function in the brain. Both pre- and post-ischemia intracerebroventricular PACAP38 treatment have been proven to attenuate the global and focal ischemia-induced brain damage [43, 44, 114, 141, 152, 157, 171]. Both continuous infusion and single injection was used in pre- or post-ischemia treatment. Pre-ischemia continuous infusion was usually started 48h before ischemia with a small dosage and rate (1pmol/L) [43, 44], which allowed the full distribution of PACAP38 within the brain and CSF. A pre-ischemia single injection was regularly performed a few minutes before ischemia. However, the dosage ranged from 0.2-10 nmol per rat in different studies [96, 114, 141]. For the post-ischemia treatment, PACAP38 was generally injected immediately after ischemia with a small dosage of 1 pmol per mouse [152, 153]. Continuous infusion with 1pmol/L for rats and a single injection with 40 pmol per mouse were also proven to be neuroprotective. Recently, a novel approach demonstrated that delayed PACAP38 delivery by PACAP-producing stem cells can also promote infarct zone repair [154], which broadens the time window of intracerebroventricular PACAP38 treatment.

As a systemic peptide, intravenous injection of PACAP may have great importance, not only because of its capacity to pass the BBB, but also because of its capability of suppressing the systemic inflammatory response [153]. Intravenous injection was regularly used after ischemia with both single bolus injection and continuous infusion, which avoided the problem of PACAP degrading rapidly within the systemic circulation [172]. The single bolus injection dosage ranged from 5-20 nmol/kg, and the continuous infusion rate ranged from 16-160 pmol/h in rats [15, 98, 99]. Interestingly, the single injection with 30ng/kg PACAP38 was proven to function as a neuroprotective agent in the rat focal ischemia model [156]. The intraperitoneal single injection after ischemia was also shown to be effective in delivering PACAP38 to the brain, and further attenuated the ischemia [93].

In addition, it should be mentioned that although the intranasal administration has not been used for investigating the therapeutic value of PACAP on stroke, the intranasal administration is still a recommended method for PACAP delivery. Intranasal administration is considered a non-invasive, easily operated, and effective method for cerebral drug delivery, which can easily cross the BBB and reach a high concentration in the brain [169]. Meanwhile, current animal studies of Alzheimer's disease [173] and Huntington’s disease [85] have already presented the therapeutic potential of intranasally administered PACAP.

## Conclusion and future perspectives

In conclusion, PACAP, which mainly acts through the PAC1 receptor, exerts neuroprotective effects against ischemic brain injury *in vitro* and *in vivo*. It bears potential as a new therapeutic strategy for stroke by targeting the pathophysiological processes of ionic homeostasis, excitotoxicity, cell edema, oxidative stress, neuro-inflammation, and cell death. However, the role of PACAP in hemorrhagic stroke, and the specific underlying mechanisms need to be further explored. In addition, a better understanding of the upstream signaling components responsible for the upregulation of endogenous PACAP levels in the brain, and a further investigation of the role of VPAC1 and VPAC2 in PACAP-involved neuroprotection could help identify other effective therapeutic targets. Lastly, future well-designed preclinical experiments are necessary to optimize therapeutic regimens of PACAP. This includes investigations to determine optimal windows of treatment, dosage, and routes of administration, as well determining whether there is any potential gender- or age-related differences, which would facilitate the clinical translation of PACAP treatment in stroke patients.
